# Supplementary material for: School-based universal mental health promotion intervention for adolescents in Vietnam: Two-arm, parallel, controlled trial
Source: Glob Ment Health (Camb). 2023 Oct 23;10:e69. doi: 10.1017/gmh.2023.66 (PMC10643236; doi:10.1017/gmh.2023.66)
Supplement: Tran et al. supplementary material [file S2054425123000663sup001.docx]

Table 1. Structure of RAP-A and the Happy House program

| **RAP-A** | | **Happy House** | |
| --- | --- | --- | --- |
| **Session** | **Topic** | **Session** | **Topic** |
| 1 | Getting to know you! | 1 | Part 1: Getting to know you!  Part 2: Feeling good about yourself! |
| 2 | Building self esteem | 2 | Part 1: Introduction to the HH model  Part 2: Keep calm |
| 3 | Introduction to the RAP model | 3 | Part 1: Self talk  Part 2: Helpful thinking |
| 4 | Keep calm | 4 | Part 1: Finding solutions to problems  Part 2: Identifying and accessing support networks |
| 5 | Self talk | 5 | Part 1: Considering the other person’s perspective  Part 2: Keeping the peace and making the peace |
| 6 | Thinking resourcefully | 6 | Putting it all together |
| 7 | Finding solutions to problems |  |  |
| 8 | Identifying and accessing support networks |  |  |
| 9 | Considering the other person’s perspective |  |  |
| 10 | Keeping the peace and making the peace |  |  |
| 11 | Putting it all together |  |  |

Table 2. Overview of session content

| **Session** | **Topic** | **Key message** | **Content** |
| --- | --- | --- | --- |
| 1 | Part 1: Getting to know you!  Part 2: Feeling good about yourself! | We’re interested in you! Let’s work together as a team.  I’m OK. I’m building on my strengths. | Introduce the program.  Use fun-based activities to build rapport and gauge participant’s interactive capacity and turn-taking ability.  Introduction to the importance of regulating self-esteem. Students are introduced to a tool to monitor self-esteem.  Exploration of strengths and resources using concrete prompts. |
| 2 | Part 1: Introduction to the HH model  Part 2: Keep calm | Our body clues and our self-talk affect the way we feel and behave.  Be a detective. Find your body clues and keep calm. | Introduce HH model.  Provide a CBT based description of the link between behaviour, body clues, self-talk and emotions.  Explore body signals related to positive and negative feelings.  Develop strategies to relax and manage stress and anger. |
| 3 | Part 1: Self talk  Part 2: Helpful thinking | I am what I think.  You can change your thinking. | Cognitive restructuring with the assistance of a movie clip.  Develop positive self-talk.  Understand and identify self-talk.  Practice challenging negative self-talk and developing positive self-talk. |
| 4 | Part 1: Finding solutions to problems  Part 2: Identifying and accessing support networks | There are solutions to my problems.  There is always help at hand. | Learn problem-solving with step-by-step problem solving model to generate, carry out and evaluate solutions.  Use fun-based activities to learn about developing a support network, and seeking help to maintain emotional wellbeing. |
| 5 | Part 1: Considering the other person’s perspective  Part 2: Keeping the peace and making the peace | There are two sides to every story.  Take time out, stop and think.  Keep the peace and make the peace. | Develop perspective taking and empathy for others.  Develop strategies to prevent escalation of conflict.  Develop strategies to promote harmonious relationships and to get connected.  Develop strategies to prevent or manage conflict with care givers and significant others. |
| 6 | Putting it all together | Being a resourceful adolescent really works! Let’s celebrate! | Review program content, evaluate program, deliver personal positive feedback, and celebrate. |

Table 3. Baseline characteristics by missing the main outcome.

|  | **Complete**  (N=1,045) | **Missing**  (N=20) | **P-value** |
| --- | --- | --- | --- |
| Trial arm, n (%) |  |  | 0.07 |
| Control | 538 (97.46) | 14 (2.54) |  |
| Intervention | 525 (98.87) | 6 (1.13) |  |
| Age in years, Mean (SD) | 15.3 (0.29) | 15.3 (0.07) | 0.844 |
| Sex, n (%) |  |  | 0.329 |
| Female | 417 (97.66) | 10 (2.34) |  |
| Male | 646 (98.48) | 10 (1.52) |  |
| Mother’s education level, n (%) |  |  | 0.147 |
| *University or above* | 352 (97.24) | 10 (2.76) |  |
| *Diploma/technical degree* | 114 (100) | 0 (0) |  |
| *High school (year 12)* | 192 (98.97) | 2 (1.03) |  |
| *Secondary school (year 9) or lower* | 207 (99.04) | 2 (0.96) |  |
| *Do not know* | 198 (97.06) | 6 (2.94) |  |
| Father’s education level, n (%) |  |  | 0.483 |
| *University or above* | 350 (98.04) | 7 (1.96) |  |
| *Diploma/technical degree* | 86 (96.63) | 3 (3.37) |  |
| *High school (year 12)* | 195 (99.49) | 1 (0.51) |  |
| *Secondary school (year 9) or lower* | 180 (98.36) | 3 (1.64) |  |
| *Do not know* | 252 (97.67) | 6 (2.33) |  |
| Centre for Epidemiologic Studies Depression Scale Revised score, Mean (SD) | 11.4 (12.0) | 10.6 (12.0) | 0.772 |

Table 4. Intracluster correlation coefficient (ICC) by outcomes

|  | ICC |
| --- | --- |
| **Primary outcome** |  |
| Centre for Epidemiologic Studies Depression Scale Revised score ≥ 16 |  |
| Post-intervention | 0·027 |
| Six-month follow-up | 0·018 |
| Centre for Epidemiologic Studies Depression Scale Revised score |  |
| Post-intervention | 0·015 |
| Six-month follow-up | 0·004 |
| **Secondary outcomes** |  |
| Mental Health Continuum Short Form score |  |
| Post-intervention |  |
| Emotional well-being score | <0·001 |
| Social well-being score | 0·002 |
| Psychological well-being score | 0·009 |
| Six-month follow-up |  |
| Emotional well-being score | <0·001 |
| Social well-being score | <0·001 |
| Psychological well-being score | 0·002 |
| Coping Self-Efficacy Scale (CSES) score |  |
| Post-intervention |  |
| Problem-focused sub-scale score | 0·020 |
| Emotion-focused sub-scale score | 0·010 |
| Social support sub-scale score | 0·018 |
| Six-month follow-up |  |
| Problem-focused sub-scale score | 0·021 |
| Emotion-focused sub-scale score | 0·018 |
| Social support sub-scale score | 0·014 |
| School Connectedness Scale score |  |
| Post-intervention | 0·021 |
| Six-month follow-up | 0·044 |
